# Bitter receptor TAS2R138 facilitates lipid droplet degradation in neutrophils during *Pseudomonas aeruginosa* infection

**DOI:** 10.1038/s41392-021-00602-7

**Published:** 2021-06-04

**Authors:** Qinqin Pu, Kai Guo, Ping Lin, Zhihan Wang, Shugang Qin, Pan Gao, Colin Combs, Nadeem Khan, Zhenwei Xia, Min Wu

**Affiliations:** 1 Department of Biomedical Sciences, School of Medicine and Health Sciences, University of North Dakota, Grand Forks, ND USA; 2 Department of Neurology, University of Michigan, Ann Arbor, MI USA; 3 Wound Trauma Medical Center, State Key Laboratory of Trauma, Burns and Combined Injury, Daping Hospital, Army Medical University, Chongqing, China; 4 Biological Science Research Center, Southwest University, Chongqing, 400716 China; 5 West China School of Basic Medical Sciences & Forensic Medicine, Sichuan University, Chengdu, Sichuan China; 6 State Key Laboratory of Biotherapy and Cancer Center, West China Hospital, Sichuan University, and Collaborative Innovation Center for Biotherapy, Chengdu, Sichuan China; 7 Department of Pediatrics, Ruijin Hospital Affiliated to Shanghai Jiao Tong University School of Medicine, Shanghai, People’s Republic of China

**Keywords:** Infection, Inflammation, Infectious diseases

## Abstract

Bitter receptors function primarily in sensing taste, but may also have other functions, such as detecting pathogenic organisms due to their agile response to foreign objects. The mouse taste receptor type-2 member 138 (TAS2R138) is a member of the G-protein-coupled bitter receptor family, which is not only found in the tongue and nasal cavity, but also widely distributed in other organs, such as the respiratory tract, gut, and lungs. Despite its diverse functions, the role of TAS2R138 in host defense against bacterial infection is largely unknown. Here, we show that TAS2R138 facilitates the degradation of lipid droplets (LDs) in neutrophils during *Pseudomonas aeruginosa* infection through competitive binding with PPARG (peroxisome proliferator-activated receptor gamma) antagonist: *N*-(3-oxododecanoyl)-l-homoserine lactone (AHL-12), which coincidently is a virulence-bound signal produced by this bacterium (*P. aeruginosa*). The released PPARG then migrates from nuclei to the cytoplasm to accelerate the degradation of LDs by binding PLIN2 (perilipin-2). Subsequently, the TAS2R138–AHL-12 complex targets LDs to augment their degradation, and thereby facilitating the clearance of AHL-12 in neutrophils to maintain homeostasis in the local environment. These findings reveal a crucial role for TAS2R138 in neutrophil-mediated host immunity against *P. aeruginosa* infection.

## Introduction

Taste is one of the most important sensing functions in mammals, among which bitterness is one of the five basic tastes. A mild bitter taste produces an unpleasant sensation, while a strong bitter taste causes nausea, vomiting, and physical disgust. Therefore, the perception of bitter taste is one of the body’s effective self-protection mechanisms to prevent the intake of harmful substances.^1^ It was thought that some people are particularly sensitive to bitterness because they have more taste buds on their tongues than others. Studies have also demonstrated that sensitivity to bitterness is associated with genetic mutations in the bitter taste receptor in humans.^2^ As both human and mouse genomes contain pairs of orthologous bitter receptor genes that are conserved throughout evolution,^3^ studies on mice have been done to model taste responses elicited by stuff that taste bitter to humans for decades.^3,4^ The bitter taste receptor is a family of G-protein-coupled receptors, including 25 members, which mediate bitter signal transduction by the coupling protein, effector enzyme, and the second messenger and other factors.^5^ Recently, one of the family members—mouse bitter taste receptor type-2 member 138 (TAS2R138, homolog of human TAS2R38 (T2R38)) was reportedly associated with various physiological and disease conditions, including innate immunity,^6^ obesity,^7^ and cystic fibrosis^8^ in addition to taste sensing.^9^ In particular, *Tas2r138* is reportedly involved in immune modulation, especially airway immunity, but the underlying mechanism remains to be revealed. Recently, *Tas2r138* was found in lipid droplets (LDs) in pancreatic cancer^10^ and was a sensor of *P. aeruginosa* quorum-sensing system.^11,12^ Given the previous evidence that lipid rafts and lipids are relevant to bacterial infection and membrane process,^13,14^ we hypothesized that *Tas2r138* might be involved in LDs’ metabolism during bacterial infection.

LDs are not a simple energy reservoir in a cell, rather a complex, active, and dynamic multifunctional organelle. LDs can migrate along the cytoskeleton to interact with other organelles, and may play an important role in lipid metabolism and storage, membrane transport, protein degradation, and cell signaling.^15^ In addition, studies have shown that a variety of metabolic diseases, such as obesity,^16^ fatty liver,^17^ cardiovascular disease^18^ and diabetes,^19^ neutral lipid storage disease, and Niemann Pick C disease,^20^ are often accompanied by abnormalities in lipid storage. Therefore, research into LDs biology and function has received increasing attention.^21^ The growth of LDs is mainly controlled by two pathways: accumulation of neutral lipids and atypical LDs mediated by the CIDE (cell death-inducing DFFA (DNA fragmentation factor subunit alpha)-like effector) family proteins. Smaller LDs are mainly hydrolyzed by neutral hydrolases and acid hydrolases in lysosomes,^22^ and the catabolism of LDs is important for cell homeostasis. Perilipin-2 (PLIN2) is the well-characterized protein that localizes in the LDs and helps LDs enter into lysosomes through chaperone-mediated autophagy (CMA), dependent on PLIN2–heat shock cognate 71 kDa protein (HSC70)–lysosome-associated membrane protein 2 (LAMP2).^23^

As the human T2R38 is also shown to be colocalized in LDs in human cells,^12^ we hypothesized that mouse TAS2R138 may facilitate LDs hydrolyzation by lysosomes during *P. aeruginosa* infection in mouse cells or in mice. After detecting the *Tas2r138* expression and distribution upon *P. aeruginosa* infection, we showed that mRNA and protein levels of TAS2R138 were increased, and the protein was colocalized with LDs. Interestingly, we found that LDs were decreased after infection, raising the possibility that TAS2R138 may mediate LD degradation. Further testing demonstrated that TAS2R138 did not bind LAMP2 or PLIN2, while bound to the PPARG antagonist, acyl-homoserine lactone (AHL)-12, a mass communication molecule (autoinducer) of *P. aeruginosa* quorum-sensing system. PPARG was implicated in lipid circulation regulation.^24,25^ As both TAS2R138 and PPARG can bind AHL-12,^12,26^ we speculate that there is competitive binding for these two proteins with AHL-12. In addition, Kang et al. found that PPARG can bind to the promotor of PLIN2 to regulate the latter’s transcription.^27^ In this work, we found that bacterial infection facilitated PPARG–PLIN2 binding. After blocking PPARG by siRNA, the reduction in LDs was significantly restored to close the normal levels. As TAS2R138 is localized in the LDs, the binding with AHL-12 and the reduction of LDs could ultimately lead to the decrease in AHL-12, suggesting an association of LDs with TAS2R138. Overall, these findings identify a previously unrecognized function of mouse TAS2R138 in LD degradation and host immunity against infection, which adds a therapeutic target for antibacterial defense.

## Results

### TAS2R138 increases in alveolar macrophages (AMs) and neutrophils during *P. aeruginosa* infection

**Fig. 1:**
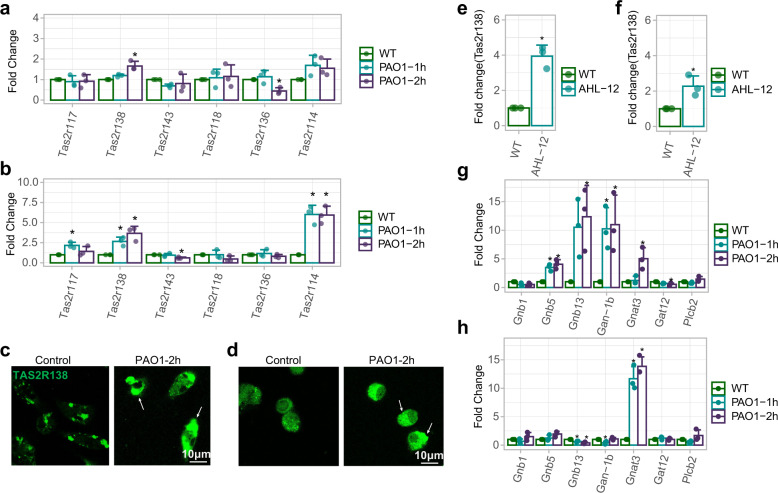
Tas2r138 increased after *P. aeruginosa* infection. AMs and neutrophils were isolated from C57BL/6N mice and infected with PAO1 (MOI: 5:1) for 1 or 2 h. Total RNA was extracted for reverse transcription and qPCR. **a**–**d** The Tas2r family mRNAs were expressed as the fold difference to GAPDH in neutrophils (**a**) and AMs (**b**), and the protein expressions in neutrophils and AMs were exhibited by confocal microscopy imaging (**c**, **d**) (arrows showing the increased TAS2R138). **e**, **f** AMs and neutrophils were infected with AHL-12 (50 µM) for 2 h. The *Tas2r138* mRNAs were expressed as the fold difference to GAPDH in neutrophils and AMs. **g**, **h** The G-protein family mRNAs were expressed as the fold difference to GAPDH in neutrophils and AMs. The results were expressed as the mean ± SD and the significant difference level between two groups was defined by one-way ANOVA with Tukey post hoc tests, *p < 0.05 (control short as CT in all figures)

AMs and neutrophils are among the highest proportions of migratory phagocytic cells in airspaces of the lung during bacterial infection. To determine whether bitter receptors play a role in bacterial infection, we detected mRNA levels of several bitter receptors whose orthologs in human were reportedly associated with airway immunity^6,28,29^ by real-time quantitative PCR (qPCR). The data showed that *Tas2r138* was the most highly expressed receptor in neutrophils (Fig. 1a), and both *Tas2r138* and *Tas2r114* were highly expressed in AMs (Fig. 1b) upon *P. aeruginosa* infection, which is in good agreement with its protein expressions determined by immunofluorescence staining (Fig. 1c, d). As AHL-12 was shown to be a sensor of T2R38 in human neutrophil cells,^12^ we speculate that this sensing may also be exerted by mouse neutrophils and hence went to examine *Tas2r138* mRNA expression after AHL-12 stimulation. The data showed that *Tas2r138* was increased in primary mouse neutrophils (Fig. 1e) and to less extent in AMs (Fig. 1f), which guides us to focus on neutrophils in following analyses. Previous work has found that there are functional interactions between type-2 bitter taste receptor (Tas2rs) and G-protein α subunits.^30^ To test whether G-protein subunits also contribute to immune responses during *P. aeruginosa* infection, we performed qPCR to analyze the expression of all known α, β, and γ subunits and their variants. The results showed increased *Gnb5*, *Gnb13*, and *Gan-1b* levels in neutrophils (Fig. 1g), while only *Gnb5* was found to be increased in AMs (Fig. 1h). These data suggest that TAS2R138 signaling in neutrophils may be more active than that in AMs. Collectively, our data delineate that TAS2R138 increases after *P. aeruginosa* infection or AHL-12 stimulation in both AMs and neutrophils, although a more marked increase in the latter.

### LDs decrease in neutrophils after *P. aeruginosa* infection

**Fig. 2:**
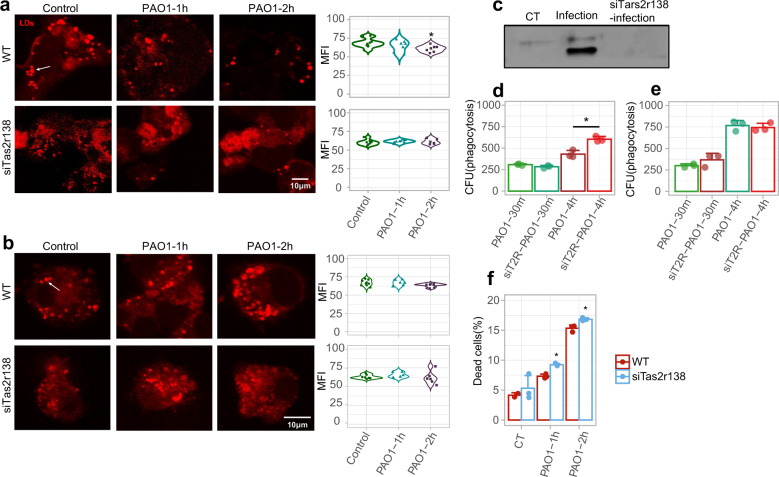
TAS2R138 was involved in LDs reduction. WT or *Tas2r138* siRNA-transfected AMs and neutrophils were infected with PAO1 (MOI: 5:1) for 2 h. **a**, **b** The LDs (stained by Nile red) expression was detected by immunofluorescence imaging in neutrophils (**a**) (arrows showed the typical LDs) and AMs (**b**). The mean fluorescence intensity (MFI) form 6–10 images were counted on the right by Image J. **c** The LDs were isolated from WT or infected and *Tas2r138* siRNA-transfected neutrophils and the TAS2R138 on the LDs was detected by western blotting. **e**, **f** AMs and neutrophils were infected with PAO1 (MOI: 5:1) for 30 min or 4 h. The phagocytosis and clearance of PAO1 were counted by CFU. WT or *Tas2r138* siRNA-transfected neutrophils were infected with PAO1 (MOI: 5:1) or AHL-12 (100 µM) stimulated for 2 h. The dead cells were stained by propidium iodide and counted by flow cytometry. The results were expressed as the mean ± SD and the significant difference level between two groups was defined by one-way ANOVA with Tukey post hoc tests, *p < 0.05

Given that *Tas2r138* expresses on the surface of LDs in human neutrophils,^12^ we used immunostaining to examine the possible colocalization of LDs and TAS2R138 (Supplementary Fig. 1a). After infection, LDs showed a reduction in neutrophils while not in the AMs. Next, we used siRNA strategy to knockdown *Tas2r138* (siT2R), and found that silencing of *Tas2r138* interrupted the reduction of LDs in neutrophils, while scrambled control RNA-siNC did not, indicating that LDs reduction during infection is TAS2R138 dependent in neutrophils but not in AMs (Fig. 2a, b and Supplementary Fig. 1b). We then isolated LDs to determine whether TAS2R138 expresses on the LDs surface, which showed that TAS2R138 expressed in the LDs derived from neutrophils (Fig. 2c). We also found that TAS2R138 did not influence the phagocytosis and clearance of bacteria in neutrophils, but showed more bacterial remnant in AMs upon 4-h infection after *Tas2r138* siRNA transfection (Fig. 2d, e). Next, we investigated whether TAS2R138 regulates the fate of neutrophils, with a focus on the function of TAS2R138 in mediating LD reduction in neutrophils. Cell viability analysis showed that silencing TAS2R138 led to an increase in dead neutrophils (Fig. 2f), suggesting that TAS2R138 in neutrophils is protective against *P. aeruginosa* infection. Altogether, these data indicate that TAS2R138 mediates LD reduction to protect neutrophils from bacterial attack.

### TAS2R138 facilitates LDs entering into lysosomes and subsequent degradation

**Fig. 3:**
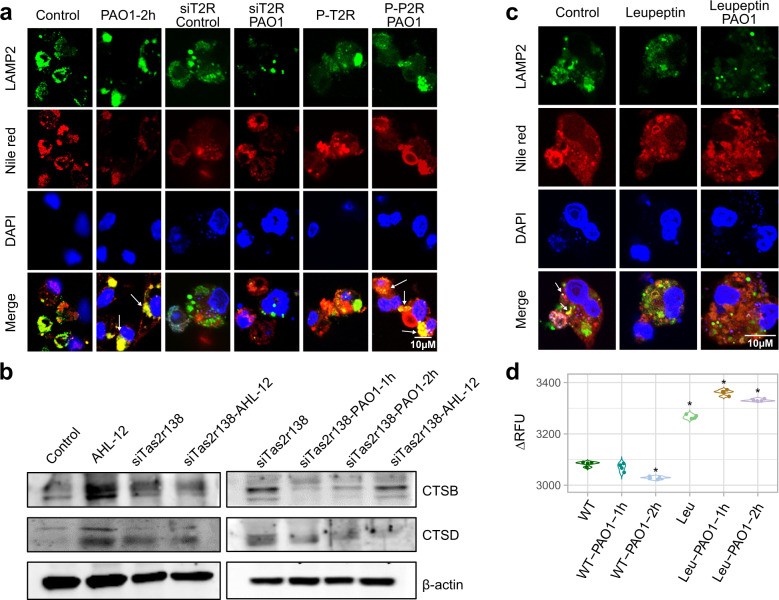
LDs entered into lysosomes. **a** WT or *Tas2r138* siRNA-transfected neutrophils were infected with PAO1 (MOI: 5:1) or AHL-12 (50 μM) stimulated for 2 h. The LDs (stained by Nile red) and LAMP2 (green) were detected by immunofluorescence imaging (arrows showing the typical proteins colocation). **b** WT or *Tas2r138* siRNA-transfected neutrophils were infected with PAO1 (MOI: 5:1) or AHL-12 (50 µM) stimulated for 2 h. The expression of CTSB, CTSD, and β-actin was tested by western blotting. **c** Neutrophils were incubated with 20 µM leupeptin for 24 h to inhibit lysosome function. Then the cells were infected with PAO1 (MOI: 5:1) for 2 h. The LDs (stained by Nile red) and LAMP2 (green) expression were detected by immunofluorescence imaging (arrows show the colocation between LAMP2 and LDs decrease). **d** Neutrophils were incubated with 20 µM leupeptin for 6 h, then the cells were infected with PAO1 (MOI: 5:1) for 2 h. Cells were fixed by 4% paraformaldehyde and permeabilized with 0.2% Triton-X 100 for 15 min. The LDs were stained by Nile red for 2 h. After washed by PBS, the fluorescence intensity was measured by Bio-Tek fluorescence plate reader (530/25, 590/35). The results were expressed as the mean ± SD and the significant difference level between two groups was defined by one-way ANOVA with Tukey post hoc tests, *p < 0.05

To track the dynamic trafficking of LDs, we detected the colocalization of LDs with lysosomes. The results showed colocalization between LAMP2 and LDs (Fig. 3a), as well as a colocalization between LAMP1 and LDs (Supplementary Fig. 2), while silencing of *Tas2r138* dampened the colocalization. Then, we analyzed the lysosome function by detecting the lysosome proteins cathepsin B (CTSB) and cathepsin D (CTSD) via western blotting, and found that CTSB and CTSD were increased after infection, which was almost abolished by *Tas2r138* knockdown (Fig. 3b). By blocking lysosome function with leupeptin (lysosome inhibitor), we observed increased LDs and enhanced fluorescence intensity after infection compared to the no inhibitor group (Fig. 3c, d). Collectively, these results illustrate that TAS2R138 may mediate LD degradation in the lysosome compartment of neutrophils.

### TAS2R138 accelerates PPARG redistribution and binding with PLIN2

**Fig. 4:**
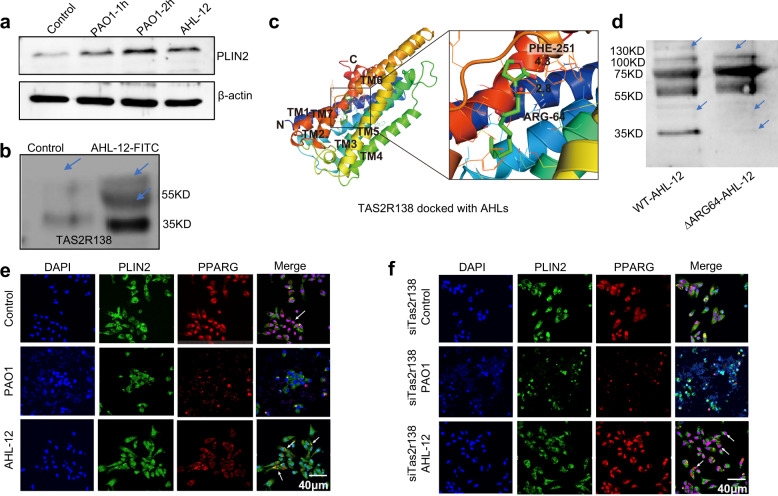
Tas2r138 competitively bound to antagonists of PPARG. **a** To assess potential interaction between AHL and TAS2R138, WT or *Tas2r138* siRNA-transfected neutrophils were infected with PAO1 (MOI: 5:1) or AHL-12 (50 µM) stimulated for 2 h. The LDs (stained by Nile red) and PLIN2 (green) expression were detected by western blotting (arrows showing the binding bands). **b** Dynabeads were incubated with the TAS2R138 antibody overnight at cells 4 °C and decant by the magnetic stand. Neutrophils (1 × 10^7^) were lysed with RIPA and protease inhibitor cocktail and incubated with beads overnight at 4 °C with or without AHL-12-FITC (300 µM). Then the proteins conjugated with beads were isolated by the magnetic stand and measured by western blotting. **c** The structure of TAS2R138 was modeled by the Swiss model: https://swissmodel.expasy.org/interactive/?ac=Q7TQA6, and the docking analysis was performed on AutoDock platform. **d** WT or R64A point mutation plasmids were transfected to neutrophils for 24 h. Cell lysate was co-cultured with AHL-12 and TAS2R138 was detected by western blotting (arrows showing the binding bands). **e**, **f** WT or *Tas2r138* siRNA-transfected neutrophils were infected with PAO1 (MOI: 5:1) or AHL-12 (50 μM) stimulated for 2 h. The PPARG (red) and PLIN2 (green) expression was detected by immunofluorescence imaging (arrows showing typical nuclear distribution and cytoplasmic distribution of PPARG)

One way for LDs entering into the lysosome is the molecular CMA. LD surface protein PLIN2 binds LAMP2 via the HSP70 chaperone.^15^ Herein, we hypothesized that TAS2R138 enhances the LD degradation by increasing PLIN2 expression or by potentiating the PLIN2’s binding to lysosomes. Using immunofluorescence (Supplementary Fig. 3a) and western blotting analysis (Fig. 4a), we observed that PLIN2 increased after infection or AHL-12 treatment. As prior studies showed that AHL-12 could bind with TAS2R138 in human leukemia cells^11^ as an antagonist of PPARG, we speculated that TAS2R138 competitively binds with AHL-12 to release PPARG. To test this possibility, we pulled down TAS2R138 (using beads) with AHL-12-FITC,^11^ and confirmed that AHL-12 could also bind TAS2R138 in mouse neutrophils (Fig. 4b). In addition, the affinity of AHL-12 to TAS2R138 was tested to be 4 µM (Supplementary Fig. 3b). To determine the binding site, we used the SWISS-MODEL Interactive Workspace for modeling the structure of TAS2R138 and docking the potential sites with AHL-12. The docking analysis showed that the mode of interaction between this compound (AHL-12) and TAS2R138 possibly through arginine 64 site (R64) and phenylalanine site 251 of this protein (Fig. 4c). To further assess the function of the binding, we generated R64A point mutant plasmid and used it to transfect neutrophils. The cell lysate was co-cultured with AHL-12 and TAS2R138 protein was determined by western blotting. The results showed that there was reduced binding compared to the WT plasmid-transfected control, suggesting that R64 is important for AHL-12–TAS2R138 binding (Fig. 4d). Hence, these data confirmed the interaction between AHL-12 and TAS2R138. Using immunofluorescence, we noticed that PPARG was redistributed from nuclei to cytoplasm and colocalized with PLIN2 after infection or AHL-12 treatment (Fig. 4e), and the redistribution could be weakened by silencing *Tas2r138* (Fig. 4f). The opposite result of overexpression plasmid transfection was shown in Supplementary Fig. 3c. These data confirmed that TAS2R138 is involved in PPARG redistribution and binds with LD surface protein PLIN2, which is recognized by lysosomes.

### PPARG binds PLIN2 to facilitate PLIN2-mediated LDs degradation and AHL-12 clearance

**Fig. 5:**
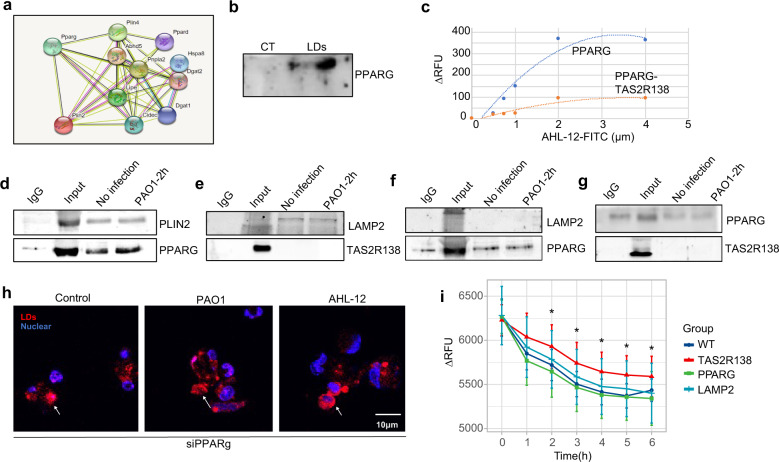
PPARG bound to LDs’ surface protein PLIN2 to facilitate lipid droplet degradation and AHL-12 clearance. **a** A potential interaction between PLIN2 and PPARG in mouse was predicted by “STRING” online on 11/19/2019. **b** LDs were isolated from normal or infected neutrophils, and PPARG on the LDs was detected by western blotting. **c** PPARG protein was coated on the plate using ELISA kit, co-cultured with different concentrations of AHL-12–FITC individually or AHL-12–FITC and TAS2R138 protein together for 2 h. The unbound AHL-12–FITC was washed out, and the fluorescence intensity was detected by a plate reader. The statistical curve corresponding to the AHL-12–FITC concentration and fluorescence value was fitted. **d**–**g** Dynabeads were incubated with antibodies (PLIN2/PPARG: PLIN2 and PPARG as the probe antibody individually to pull down each other, LAMP2/TAS2R138, LAMP2/PPARG, and PPARG/TAS2R138) individually or IgG antibody overnight at 4 °C and decant by the magnetic stand. Neutrophils were lysed and incubated with beads overnight at 4 °C. Then the proteins conjugated with beads were isolated by the magnetic stand, and corresponding proteins were measured by western blotting. **h** Neutrophils were transfected with PPARG siRNA for 24 h then infected with PAO1 or AHL-12. LDs were stained by Nile red (arrows showing the typical LDs). **i** Neutrophils cultured with or without AHL-12–FITC and TAS2R138, PPARG, or LAMP2 antibody for 2 h. The residual AHL-12–FITC was detected by Bio-Tek fluorescence plate reader (530/25,590/35). When stronger fluorescence is detected, there is more free AHL-12 in the reaction solution (due to not binding to TAS2R138). The results were expressed as the mean ± SD and the significant difference level between two groups was defined by student *t* tests, *p < 0.05

To further elucidate the molecular mechanism for the interaction between PLIN2 and PPARG, we first used the protein interaction program STRING to predict the potential binding between these two proteins. The computing results showed that PLIN2 possesses a high probability of binding PPARG (Fig. 5a). We also used an online prediction website https://genemania.org/search/homo-sapiens to predict the relationship between PLIN2 and PPARG, and the result similarly indicates that PLIN2 and PPARG may interact in human cells too (Supplementary Fig. 4a). We also used the SPRING website developed by Dr. Zhang lab to predict the interaction models^31^ (The top three rank models shown in Supplementary Fig. 4b), which renders elevated possibility of the PPARG and PLIN2 binding. Therefore, we isolated LDs and detected the existence of PPARG in the isolates, which showed the presence of PPARG in isolated LDs (Fig. 5b). This is consistent with imaging colocalization data (green-PLIN2 and red-LDs; Supplementary Fig. 5a). These data further support that PPARG can bind PLIN2. Then we tested the affinity of AHL-12–PPARG using PPARG ELISA kit. PPARG protein was coated on the plate and different concentrations of AHL-12–FITC were added to evaluate the binding ability, which showed positive binding (Fig. 5c; blue: *K*_d_ = 3.2 µM). To probe the competitive binding of PPARG to TAS2R138, we also added TAS2R138 together with AHL-12–FITC, and the data showed deceased binding between PPARG and AHL-12 in the presence of TAS2R138 (Fig. 5c) (orange: *K*_d_ = 2 µM). To ascertain the molecular interaction, we performed a series co-IP and affirmed that PLIN2 beads pulled down PPARG, and likewise, PPARG beads were also able to pull down PLIN2, strongly indicating that PLIN2 bound with PPARG (Fig. 5d). Furthermore, we found that PLIN2 also bound to LAMP2 (Supplementary Fig. 5b). However, neither TAS2R138 nor PPARG bound to LAMP2 (Fig. 5e, f). Moreover, PPARG did not bind TAS2R138 (Fig. 5g). Taken together, we concluded that PPARG specifically bound to PLIN2, while not bound to LAMP2 or TAS2R138. By siRNA silencing of PPARG, we found that LDs did not further reduce after infection or AHL-12 treatments (Fig. 5h). These data indicate that PPARG binds PLIN2 to accelerate LD degradation. Considering that TAS2R138 is also expressed on the surface of LDs, we posited that TAS2R138 might interact with AHL-12 to degrade LDs. To test the hypothesis, we added AHL-12–FITC into control neutrophils and siTas2r138-treated neutrophils for 6 h and detected FITC-positive cells by flow cytometry analysis. Our results showed that AHL-12–FITC remained at higher levels in the *Tas2r138* knocked down cells than those in WT cells (Supplementary Fig. 5c). We also used antibodies specific for T2R38, PPARG, and LAMP2 to block the cells, respectively, and then tested residual FITC fluorescence intensity. The data showed that elevated AHL-12–FITC remained after blocking T2R38, consistent with the FACS data (Fig. 5i). Collectively, these results reveal that PPARG bound with PLIN2 to facilitate PLIN2-mediated LD degradation and AHL-12 clearance.

### TAS2R138 plays a vital role in neutrophil-mediated immunity against *P. aeruginosa* in vivo

**Fig. 6:**
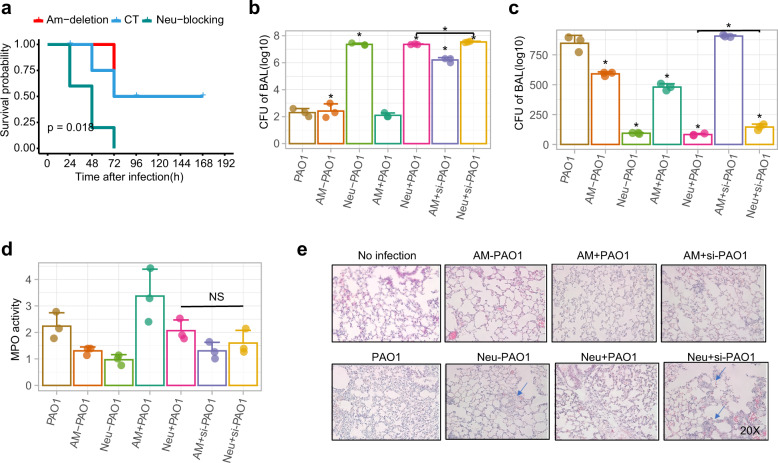
Tas2r138 played protective roles against *P. aeruginosa* infection in vivo. **a** Eight to 12 weeks male and female (sex-matched) mice were subjected to neutrophil depletion by injecting (i.p.) 0.1 mg GR-1 monoclonal antibody with rat IgG as control at 24 or 48 h before bacterial infection. AMs were depleted by i.n. instillation of 100 µl of clodronate-containing 48 h before infection with 1 × 10^7^ PAO1.^44^ Mouse survival was monitored for 7 days. Survival was represented by Kaplan–Meier curves. (*p* = 0.0273, *n* = 6, log-rank test). **b**, **c** After AMs and neutrophils depletion, 1 × 10^6^ normal or siTas2r138 AMs or siTas2r138 neutrophils were transferred by I.V. injection. **d**, **e** CFU and total cells test in BAL (B blocking, D depletion), MPO test, cytokines test (from serum), and histochemical staining were performed after 24 h post infection and euthanasia mice (blue arrows showing the 10× augmented view for indicated areas). The results were expressed as the mean ± SD and the significant difference level between two groups was defined by student *t* tests, *p < 0.05. AM− AM cell deletion, AM+ AM cell deletion and then transferred AM back, AM + si AM cell deletion and then transferred siTas2r138 AM back, Neu− Neu blocking, Neu+ Neu blocking and then transferred Neu back, Neu + si Neu blocking and then transferred siTas2r138 Neu back

LDs degradation and AHL-12 clearance are integral to maintaining homeostasis. Data in Fig. 2f showed the importance of TAS2R138 for host cell survival. To infer the physiological relevance, we tested whether the TAS2R138-mediated LD degradation occurs in vivo. Using both normal male and female C57BL/6 WT mice to compare the difference between AMs-depleted mice and neutrophil-depleted mice, which showed that depletion of neutrophils led to lower mouse survival (Fig. 6a). By adoptive transfer of normal, siTas2r138 AMs or siTas2r138 neutrophils by I.V. injection, we observed that the siTas2r138 neutrophils recipient group showed the most severe inflammation, exacerbated CFU in bronchoalveolar lavage (BAL; Fig. 6b), decreased neutrophil counts, subdued MPO (Fig. 6c, d), severe tissue injury (Fig. 6e), and heightened cytokines in the serum (Supplementary Fig. 6a–d) and tissue (Supplementary Fig. 6e–g). To reduce potential autoimmunity, we use male mice for isolating neutrophils for this experiment, as male’s immunity is less sensitive than female’s.^32^ Next, we stained LAMP2 (green) and LD (red) in the tissue, and found that after PAO1 infection, increased colocalization between LAMP2 and LDs, whereas deleting neutrophils reduced the colocalization. Furthermore, the colocalization could be reversed by adopter transfer of normal neutrophils, but not siTas2r138-transfected neutrophils (Supplementary Fig. 7). Taken together, our data indicate that the TAS2R138-mediated LD degradation is protective against PAO1 infection in vivo, suggesting an essential role of TAS2R138 in immune defense in mouse neutrophils.

## Discussion

In this study, we report that through competitive binding PPARG antagonist AHL-12, TAS2R138 plays an important role in neutrophil-mediated host immunity against *P. aeruginosa* infection by facilitating LDs degradation. The released PPARG accelerates LDs degradation and AHL-12 prolongs the survival of host cells in infection via binding PLIN2. These findings extended the roles of bitter receptor TAS2R138 in host defense in murine neutrophils and inferring a new antibacterial role in host defense. Given that TAS2R138 is just one of the members in the bitter receptors and/or taste receptors and AHL-12 is only one of the various effector factors of *P. aeruginosa*, there is a high probability that taste receptors function as the sensors of *P. aeruginosa* and potentially other QS bearing bacteria.

Here, we find that TAS2R138 not only functions as an AHL-12 sensor, but also colocalizes with LDs and plays indispensable antibacterial roles. LDs were originally thought to serve only as a fat depot and had not been identified as a highly dynamic organelle with pivotal roles in the regulation of lipid metabolism until the proteins on the LD coats were discovered in the 1990s.^33^ Gradually, LDs’ roles in inflammatory responses, obesity, and cancer have been recognized.^34–36^ Due to the complex process of lipid metabolism and multiple roles of LDs in membrane transport, protein degradation, and signaling transduction, the chapter of LDs study has suddenly been open. PPARG is one of the most studied nuclear receptor proteins that have ample roles in regulating LDs, including LDs formation,^37^ controlling lipid catabolism^38^, and LDs protein levels.^27^ We have now revealed a new function for PPARG binding with PLIN2 and accelerating LDs degradation. However, whether PPARG directly or indirectly binds with PLIN2 and how PPARG changes the PLIN2 conformation remains to be determined. PPARG, PLIN2, and TAS2R138 all have the KFERQ motif that can be recognized by a molecular chaperone and certain proteins for CMA from LDs to bind LAMP2 and ultimately degradation in the lysosome.^15^ Hence, we posit that there is a possibility that PPARG helps to expose PLIN2 KFERQ motif or PPARG helps recruit molecular chaperones. We also found that there is a binding between TASR138 and AHL-12 (Fig. 4).

**Fig. 7:**
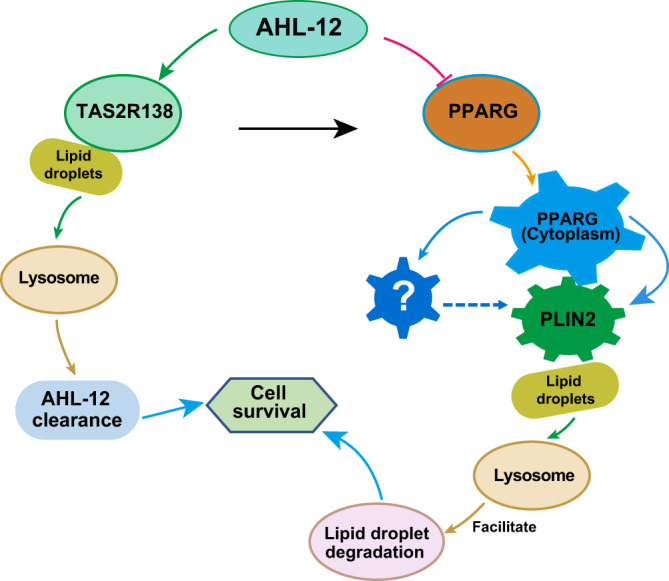
Schematic diagram of TAS2R138 involved lipid droplet degradation pathway. During *P. aeruginosa* infection, TAS2R138 competitively conjugated with PPARG antagonist AHL-12. The released PPARG redistributes from nuclear to cytoplasm to accelerate LDs degradation via binding with PLIN2. TAS2R138 binding with AHL-12 is associated with LDs, which is degraded during infection, thus helped the clearance of AHL-12 in neutrophils and promoting cell survival

AHL effector factors (C4-AHL, C8-AHL, and 3-oxo-C12-AHL) are secreted by Gram-negative bacteria and regulated by QS systems. AHL molecules have a common homoserine lactone ring head. Different AHL molecules have distinct acyl side chain tails. The differences among their structures and functions may lie in the side chain length and the substituents, which may also cause microorganisms to have certain specificity in using AHL.^39^ Jaggupilli et al. reported that Tas2r members including Tas2r4, Tas2r20, and Tas2r14 can be activated by 3-oxo-C12-AHL but not C8-AHL. Mechanistically, we speculate that AHLs bind to Tas2rs by connecting to a similar orthosteric site that is present on the extracellular surface of Tas2r.^40^ Therefore, the family of Tas2rs is important “sensors” of bacteria in host–pathogenic interplay. The clearance of AHL-12 after sensors’ activation is also important for quelling the cascade to maintain the homeostasis. Our finding that TAS2R138 helps AHL-12 clearance together with LDs degradation is crucial and timely. TAS2R138 is not only a controller of the AHL-12 activated pathway, but also an indirect activator of PPARG (Fig. 7). The in vivo data also showed the important role of TAS2R138 in neutrophil-mediated host immunity against *P. aeruginosa* infection.

Nevertheless, the functions of this bitter receptor (TAS2R138) and other related family members and their underlying mechanisms involved in LD interaction and pathogen clearance are currently unclear and needs continued research. Another limitation for this study is that a *Tas2r138* knockout mouse may help further solidify the role of TAS2R138 in host immunity against infection by bacteria or other pathogens, such as SARS-CoV-2, the causal pathogen of the COVID-19 pandemic. A minor caveat is that the markets may have many uncharacterized polyclonal antibodies. Unfortunately, there is limited characterization of the few commercial anti-TAS2R138 Abs. Further study will require well-characterized antibodies and use of mass spectrometry and other technologies to prove the binding, rigorously. In conclusion, bitter receptor TAS2R138 can be viewed as an emerging pathogenic molecular pattern receptor, which plays multiple roles during *P. aeruginosa* infection and indicate therapeutic targets for control of this or other refractory infections.

## Materials and methods

### Mice

Six to 12-week-old (female and male) C57BL/6N mice were obtained from Envigo Harlan laboratories and housed in the Center for Biomedical Research of University of North Dakota. All animal care and experimental procedures were approved by the University of North Dakota institutional animal care and use committee.^41^

### Bacterial strains and infection experiments

*P. aeruginosa* strain, PAO1 was a gift from Dr. S. Lory (Harvard Medical School). It is grown in lysogeny broth (LB) at 37 °C in 220 r.p.m. shaking speed. PAO1 was cultured overnight in LB at 37 °C with shaking (220 r.p.m.). The next day, taking 50 µl of bacterial suspension to another new 5 ml LB and cultured 2–4 h until the optical density (OD) at 600 nm was 0.6–0.8. Then the cell number was adjusted for infection (1 OD unit = 1 × 10^9^ cells/ml). Before infection, mice were randomly grouped (*n* = 6) and anesthesia by ketamine. A total of 1 × 10^7^ bacteria were intranasally (40 µl) to the mice. For cell infection: cell medium was replaced with antibiotic-free medium, and the bacterial density was adjusted just like the animal infection. AMs or neutrophils were infected (MOI) of 5:1 (bacteria–cells ratio) for 1 h or 2 h.^42,43^

### AMs depletion and neutrophils blocking

For AMs depletion, mice were i.p. instilled with 100 µl of clodronate 48 h before infection. For neutrophil blocking, we injected mice intraperitoneally with 0.1 mg of antibody (100 µl in PBS) Gr-1 (Biolegend) at 48 h before bacterial infection.^44^

### Cells

AMs were isolated from BAL the lungs for ten times with 0.50 ml 0.90% NaCl. AMs were collected by centrifugation at 300 × *g* for 10 min and cultured in 1640 + 10% FBS + 1% penicillin–streptomycin + 10 ng/ml GM-CSF. Neutrophil cells were isolated from peritoneal lavage fluid from the mice which by i.p. (intraperitoneal injection) injecting 1 ml 3% thioglycollate broth for 24 h. Cells were maintained in RPMI 1640 with 10% FBS + 1% penicillin–streptomycin + 50 ng/ml G-CSF to delay the apoptosis.^45^

### Lipid droplets isolation

The LDs were isolated from the neutrophil followed the manufacturer’s instructions (Cell Biolabs, INC. cat# MET-5011). Briefly, 1.5 × 10^7^ neutrophils were washed with PBS for three times to totally removing the media. The cell pellet was resuspended with 200 µl of reagent A. After incubating 10 min, add 800 µl of reagent B and homogenized the cells by passing the cells five times through 27-gauge needle. Then took 600 µl first layer of reagent B and centrifuged for 3 h (18,000 × *g*, 4 °C). The LDs were contained in the top 270 µl of reagent B.

### H&E staining

Lung tissues were fixed in 10% formalin (Sigma-Aldrich) for 72 h and then were infiltrated in 20% sucrose for 72 h. Tissues were embedded in OCT (optimal cutting temperature compound), cut by cryostat and stained by standard H&E protocol.^46^ The slides were imaged by Nikon Eclipse 80i microscopy by 20× objective.

### ELISA for detecting cytokines

Cytokines IL-6, IL-1β, TNF-α, and IL-22 were detected by ELISA kits (all the kits ordered from Invitrogen). Samples of serum and lung homogenates were collected at 24 h after PAO1 infection. The concentrations were measured according to the corresponding standards.^47^ The concentration of the cytokines was calculated referred to the formula based on the standard curve.

### Myeloperoxidase assay

Record the weight of each sample. Lungs were homogenized in HTAB buffer (5 g HTAB into 1 l of potassium phosphate buffer (50 mM, pH = 6.0). Then supernatants were decanted after centrifuged (13,400 × *g* at 4 °C), and 7 µl samples were added into 200 µl reaction buffer (0.167 mg/ml O-dianisidine, 50 mm KH_2_PO_4_, pH 6.0, and 0.0005% mm H_2_O_2_). Read absorbance at 450 nm at 30 s intervals. The concentration of sample A was calculated as [Δ*A*(*t* 2 − *t* 1)]/Δmin × (1.13 × 10^−2^).^48^

### Pull-down and Co-IP assays

Dynabeads (Invitrogen, sheep anti-mouse IgG cat#11031 and sheep anti-rabbit IgG cat#11203D) were incubated with TAS2R138 or PPARG antibody overnight at cells 4 °C and decant by the magnetic stand. Cells (1 × 10^7^) were lysed with RIPA and protease inhibitor cocktail buffer and incubated with beads overnight at 4 °C with or without AHL-12-FITC (300 µM). Then the proteins conjugated to beads were isolated by the magnetic stand and measured by western blotting. The co-IP assays were performed similar procedure by omitting AHL-12 and used A protein incubated beads to capture and detected corresponding B protein.^49^

### Western blotting analysis

Cells were lysed with Pierce RIPA Buffer (Thermo Fisher, cat# 89901) with protease inhibitor cocktail (Thermo Fisher, prod #1861278). The lysates were quantified by bicinchoninic acid (BCA) protein assay. Samples were separated by electrophoresis on 10–12% SDS–PAGE gels and transferred to 0.22 µm nitrocellulose filter membrane. Proteins were blocked by 5% dry milk and detected by immunoblotting using primary Abs. Following with corresponding secondary Abs and ECL reagents to detect specific interaction and specific protein.^50^ The Abs were used from different vendors as stated below: PPARG (Santa Cruz, cat# sc-7273), PLIN2 (ABclonal, cat# A6276), TAS2R138 (MyBioSource, cat# MBS8532047), CTSD (Santa Cruz, cat# sc-3741381), CTSB (Santa Cruz, cat# sc-365558), β-actin (Santa Cruz, cat# sc-4778), LAMP1 (Abcam, cat# ab25245), and LAMP2 (Abcam, cat# ABL-93-S).

### Transfection of small interfering RNA

Neutrophil cells were transfected with 50 nM Tas2r138 /PPARG or control siRNAs or full-length Tas2r138 plasmid using Lipofectamine 3000, following manufacturer’s instruction. After 24 h interfered, cells were infected with PAO1 or AHL-12 in stated time.^51^ The knocking down of Tas2r138/PPARG was confirmed by western blot or Immunofluorescence imaging.

### Immunofluorescence imaging

AMs and neutrophils were cultured in 24-well dishes with the glass cover slips on the bottom. After three times washing by PBS, cells were fixed by 4% paraformaldehyde and permeabilized with 0.1% Triton-X 100 for 15 min then blocked with 5% bovine serum albumin (BSA) overnight at 4 °C. Slides were incubated with ASC and caspase-1 antibodies (1:250 in 1% BSA in PBS) overnight at 4 °C. The cells were stained with fluorescence-conjugated secondary. After three times washing with PBS, nuclei were stained with DAPI. Slides were visualized with Olympus FV3000 microscope.^52^

### Quantitative RT-PCR

Total RNA of cells was extracted using TRIzol (Invitrogen, Life Technologies cat# 15596018), and the RNA was reversed transcription to cDNA using the High-capacity cDNA reverse transcription kit (Thermo Fisher scientific cat# 4374966), following the manufacturer’s instructions. Quantitative RT-PCR was performed by CFX Connect System (Bio-Rad). The mRNAs were expressed as the fold difference to GAPDH. The primers used in this study were showed in supplementary Table S1.^53^

### Cell survival

Neutrophils were transfected with Tas2r138 siRNA for 24 h and then were infected with PAO1 (MOI:5) for 2 h (the bacterial culturing and density calculation was the same as the infection experiments). Cell survival was tested via propidium iodide (PI) staining (40 mg/ml PI for 30 min). The PI-positive cells were measured by Flow cytometry.^54^

### Flow cytometry

Cells were transfected with Tas2r138 siRNA for 24 h and were challenged with AHL-12–FITC (Cayman Chemical) for 6 h. Then, cells were washed three times with PBS and digested with trypsin. The harvested cells were measured by Flow cytometry to count FITC-positive cells.^47^

### Affinity analysis

TAS2R138 and PPARG ELISA kits were ordered from MyBioSource. The standard proteins (PPARG: 100 μg/well, TAS2R138: 10 pg/well) were coated on the plate followed the manufacturer’s instructions. The AHL-12-FITC was co-cultured with the proteins for 2 h followed different concentrations. Then the unbound AHL-12 was washed out by corresponding washing buffer. Relative standardized fluorescent intensity (ΔRFU) was detected by a Bio-Tek fluorescence plate reader (530/25, 590/35).

### R64A mutant construction

The *Tas2r138* gene cDNA ORF clone sequences were retrieved from the NCBI Reference Sequence Database (GenBank: BC128016.1). The ORF sequences were cloned into the vector pcDNA3.1+/C-(K)DYK (clone ID: OMu58142, Genescript). The R64A mutant plasmid was amplified by Thermo Fisher Scientific Phusion high-fidelity PCR kit and ligated by GeneArt seamless cloning and assembly kit, then transformed to DH5α for plasmid application. Then the nonmutant and mutant plasmids were transfected to neutrophils. Twenty-four hours post transfection, cell lysate was qualified by BCA and co-cultured with AHL-12 for 2 h. TAS2R138 protein was detected by western blotting. CGG (R) mutant to GCG (A). Primers for mutation: mut-t2r-f, AGCATCACTGCGCTTTTCCTGCAGGGCCCTGC and mut-t2r-r, CAGGAAAAGCGCAGTGAT GCTGAGACACAGCAG.

### Molecular modeling and ligand docking

Computational docking was performed using the open-source platform AutoDock version 4.2. The crystal structure of the mouse TAS2R138(Q7TQA6) protein database was downloaded from the SwissModel. The preparation of the target protein with the AutoDock Tools involved adding all hydrogen atoms to the macromolecule, which is a step necessary for correct calculation of partial atomic charges. The AHL-12 ligands structure was downloaded from the Pubchem. Docking calculation in AutoDock was performed using the protein and the desired ligand in PDB format. Then molecular docking was done with default parameters (center_*x* = 13.661, center_*y* = 42.401, and center_*z* = 70.715) to calculate the binding energies. The final result was visualized by using PyMol (http://pymol.sourceforge.net/).

### Statistical analysis

Statistical analysis was done by Student’s *t* tests and one-way ANOVA. Data are presented as MEAN ± SD and *p* value of <0.05 was considered significant and present as *p < 0.05. Statistical analysis was performed using GraphPad Prism 7 (GraphPad Software).^55^

## Supplementary information

supplemental

## Data Availability

Data used for the current study are available from the corresponding author upon reasonable request.
