# Supplementary material for: Bitter receptor TAS2R138 facilitates lipid droplet degradation in neutrophils during Pseudomonas aeruginosa infection
Source: Signal Transduct Target Ther. 2021 Jun 4;6:210. doi: 10.1038/s41392-021-00602-7 (PMC8175399; doi:10.1038/s41392-021-00602-7)
Supplement: Supplementary file 1 — supplemental [file 41392_2021_602_MOESM1_ESM.pdf]

Supplementary Materials for

Bitter receptor TAS2R138 facilitates lipid droplet degradation in neutrophils during *Pseudomonas aeruginosa* infection

Qinqin Pu, Kai Guo, Ping Lin, Zhihan Wang, Shugang Qin, Pan Gao, Colin Combs, Nadeem Khan, Zhenwei Xia and Min Wu

Correspondence to: min.wu@und.edu

This PDF file includes:

Figures. S1 to S7

Tables S1

Supplemental figures

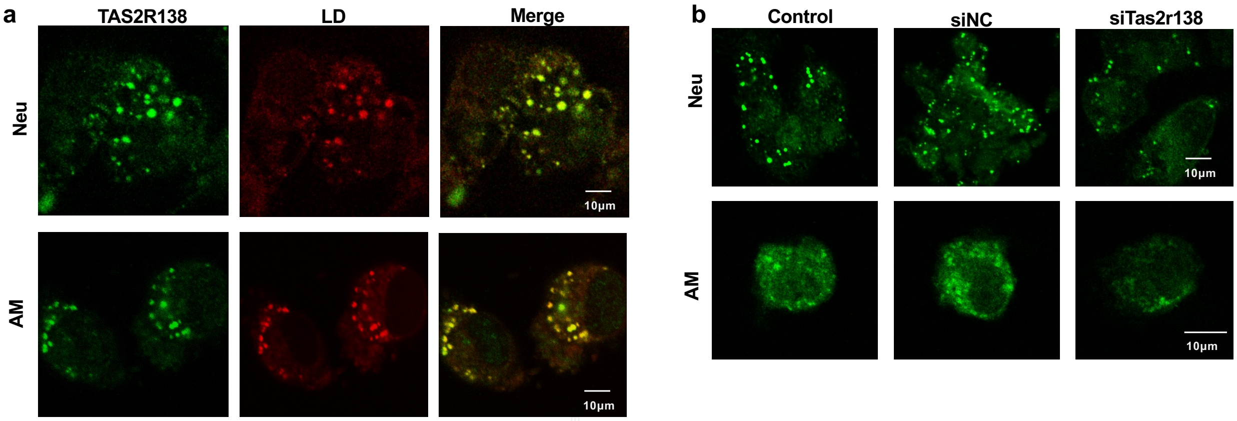

Fig. s1 LDs colocalize with Tas2r138. **a** Neutrophils or AMs were infected with PAO1 (MOI: 5:1) for 1 or 2 h. The LDs (stained by Nile red) and Tas2r138 (green) expression were detected by immunofluorescence imaging Neutrophils or AMs were transfected with control RNA (siNC) or siTas2r138 and imaged by confocal microscope. **b** The siNC did not affect theTas2r138 expression.

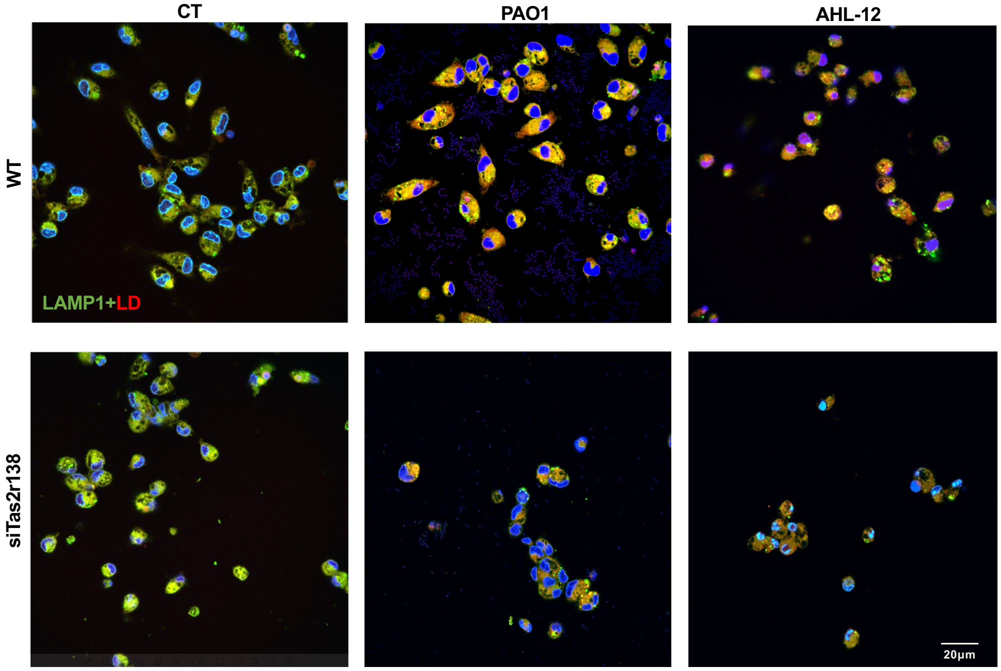

Fig. s2 LDs colocalize with LAMP1. **a** WT neutrophils or **b** *Tas2r138* siRNA-transfected neutrophils were infected with PAO1 (MOI: 5:1) for 2 h. The LDs (stained by Nile red) and LAMP1 (green) expression were detected by immunofluorescence imaging.

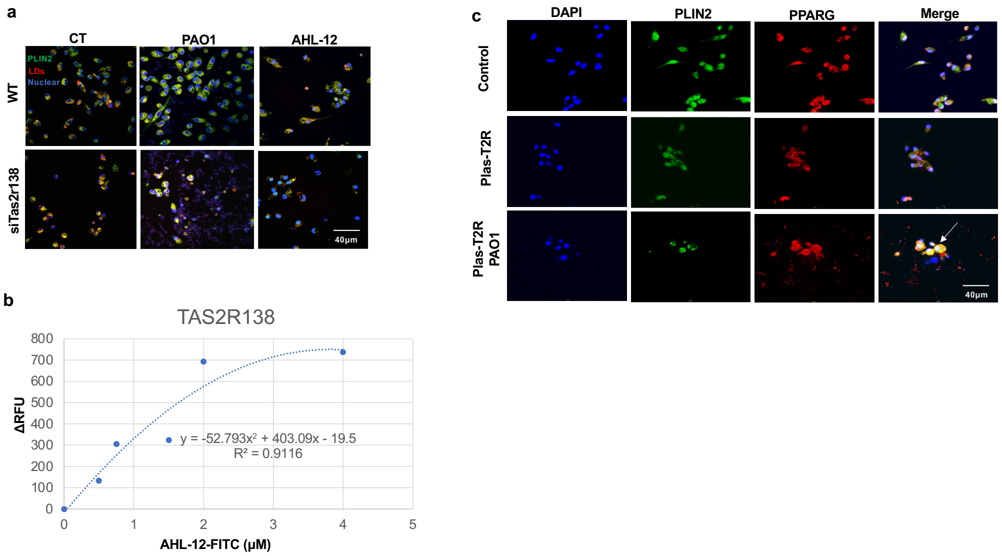

Fig. s3 LDs colocalize with PLIN2. **a** WT or *Tas2r138* siRNA-transfected neutrophils were infected with PAO1 (MOI: 5:1) or AHL-12 (50 μM) stimulated for 2 h. The LDs (stained by Nile red) and PLIN2 (green) expression were detected by immunofluorescence imaging. **b** WT or PCDNA3.1-Tas2r138 plasmid-transfected neutrophils were infected with PAO1 (MOI:5:1) stimulated for 2 h. The PPARG (red) and PLIN2 (green) expression were detected by immunofluorescence imaging. **c** The standard Tas2r138 protein (10 pg/well) was coated on the plate following the manufacturer's instructions. The AHL-12-FITC was co-cultured with the proteins for 2 h at different concentrations. Then the unbound AHL-12 was washed out by corresponding washing buffer. Relative standardized fluorescent intensity (ΔRFU) was detected by Bio-Tek fluorescence plate reader (Excitation: 530/25, Emission: 590/35). The statistical curve corresponding to the AHL-12-FITC concentration and fluorescence value was fitted.

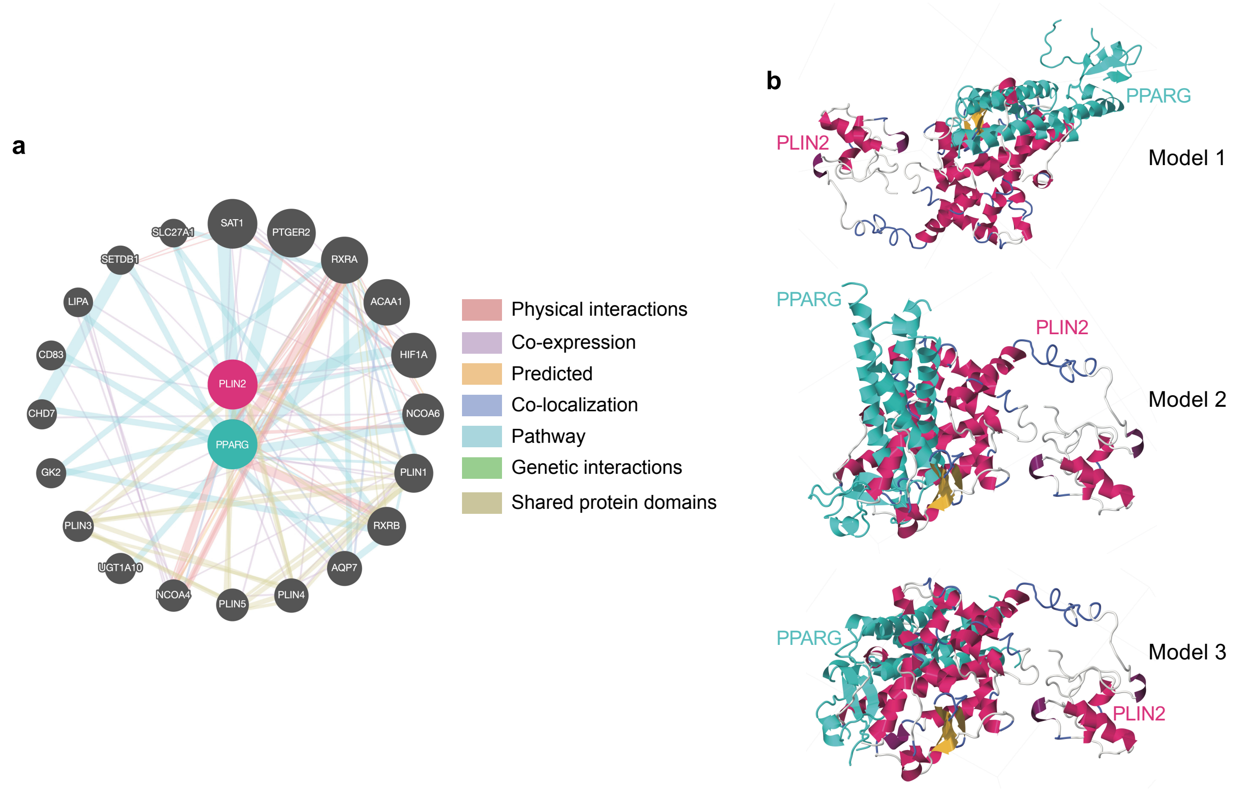

Fig. s4 Interaction prediction between PLIN2 and PPARG in human cells. **a** The potential interaction between PLIN2 (Red) and PPARG (Green) in humans was predicted by an online prediction website https://genemania.org/search/homo-sapiens. Nodes represent genes and links represent different type of interactions. Node size indicates the extent of interactions. Genes can be linked by more than one type of network. Colors stand for different types of interaction, predicted by the GeneMANIA. **b** The interaction models were predicted by SPRING: The top 3 protein complex threading results (model 1: PLIN2 (left), PPARG (right); model 2 and 3:PPARG (right), PLIN2 (left)) based on the SPRING were shown here. The SPRING score that is a combination of threading Z-score, interface contacts and TM-align match between monomer-to-dimer templates.

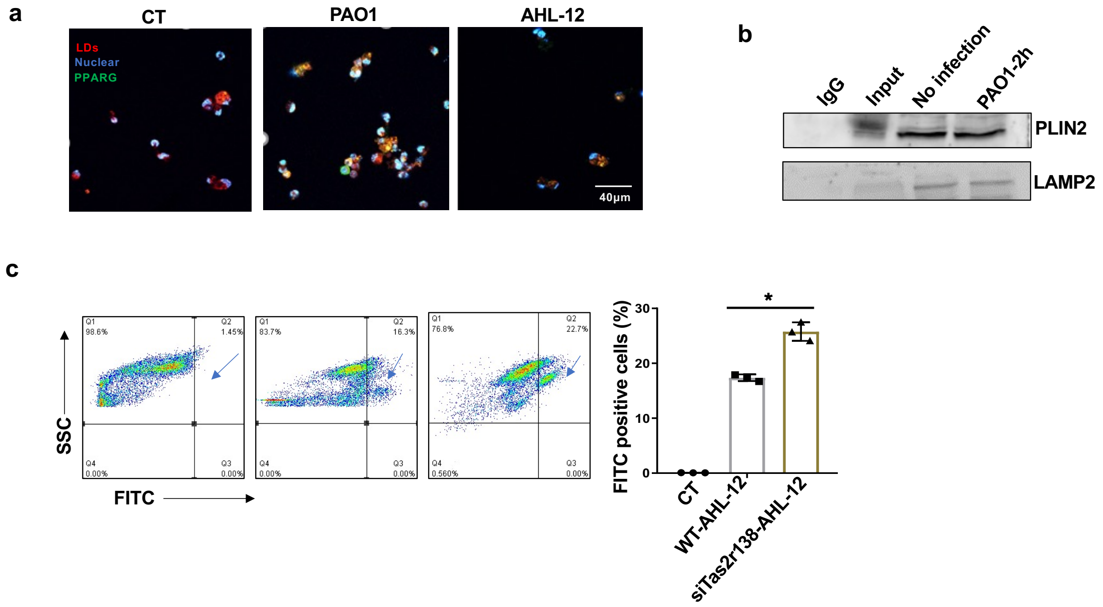

Fig. s5 PLIN2 binds to LAMP2. **a** LDs and PPARG colocalization was confirmed by immunofluorescent staining (LDs-red, PPARG-green, Blue-nuclei, merged-yellow). **b** Dynabeads were incubated with PLIN2 and LAMP2 antibodies individually or IgG antibody overnight at cells 4°C and decanted by the magnetic stand. Neutrophils were lysed and incubated with beads overnight at 4°C. Then the proteins conjugated with beads were isolated by the magnetic stand, and corresponding proteins were measured by western blotting. **c** WT or *Tas2r138* siRNA-transfected neutrophils stimulated with AHL-12-FITC (50 μM) for 6 h. The FITC positive cells were counted by flow cytometer. The results were expressed as the mean ± SD and the significant difference level between two groups was determined by student *t-tests,* *$p < 0.05$.

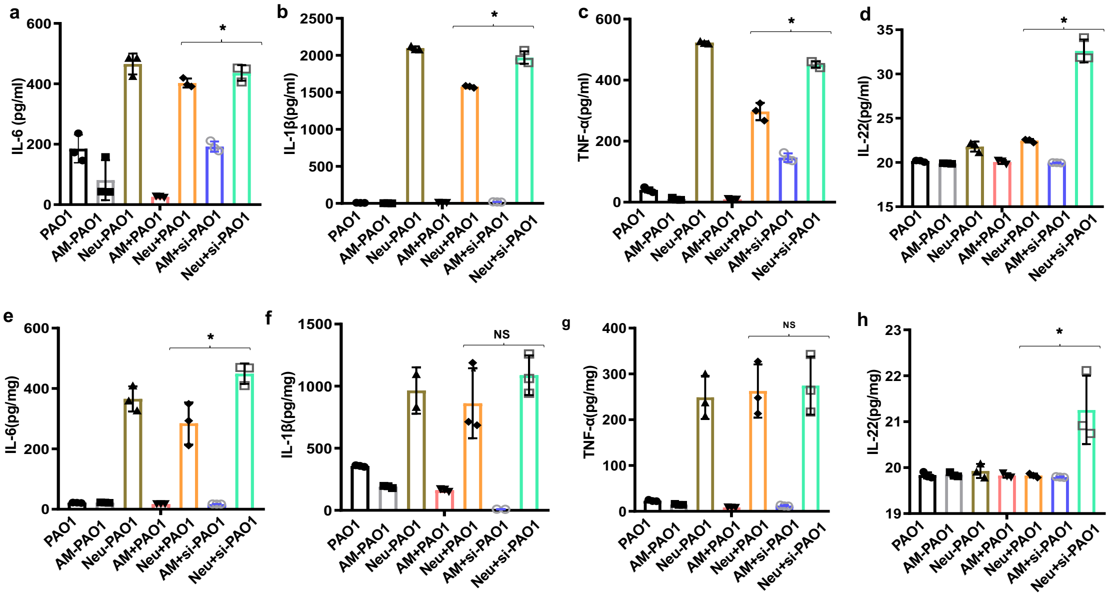

***Fig. s6 ELISA analysis of cytokines in serum and lung tissues*. a-g** 8-12 weeks' age mice were subjected to neutrophil depletion by injecting (i.p.) 0.1 mg Gr-1 monoclonal antibody with rat IgG as a control at 24 h and 48 h (total twice) before bacterial infection. AMs were depleted by i.p instillation of 100 μl of clodronate-containing 48 h before infection with 1x10^{7} PAO1. Cytokines from serum **a-c** and lungs **d-g** were detected by ELISA. The results were expressed as the mean ± SD and the significant difference level between two groups was determined by one-way ANOVA with Tukey post hoc tests, *p < 0.05.

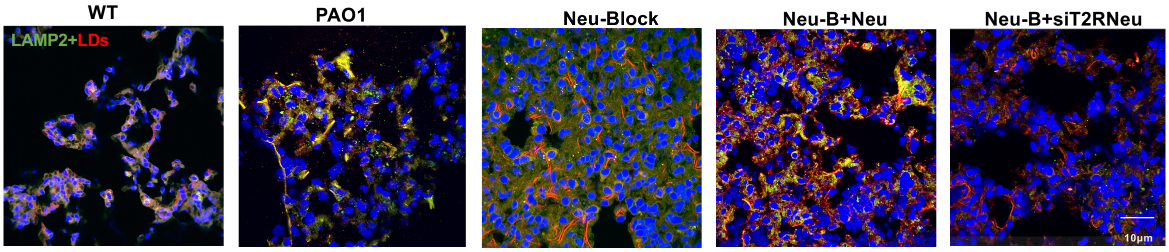

Fig. s7 Immunofluorescence staining of lung tissues. 8-12 weeks' age mice were subjected neutrophil depletion by injecting (i.p.) 0.1 mg Gr-1 monoclonal antibody with rat IgG as a control at 24 h and 48 h (total twice) before bacterial infection ($1x10^7$ PAO1). 24 h post infection, the lung was embedded in OCT and sliced for imaging LAMP2 (green) and LDs (red) (blue for nuclei) followed the immunofluorescence imaging protocol in "material and methods" section after cleaned the OCT in slides by PBS (washing 3 times).

Table s1: Primers for qPCR

Name	Sequence
Tas2r138 F	CAAACCAAGTGAGCCTCTGG
Tas2r138 R	GAGAAGCGGACAATCTTGGA
GAPDH F	AGGTCGGTGTGAACGGATTTG
GAPDH R	TGTAGACCATGTAGTTGAGGTCA
Tas2r118 F	CACTGGGTGCAGATGAAACA
Tas2r118 R	CTTCAGAACAGTGAACTGAGCTTT
Tas2r117 F	CCCTGTGGACACATCACAAG
Tas2r117 R	TCACAGTTTGTAGGGCTTTGAA
Tas2r136 F	GGACAATGAGGCTTTATGGAA
Tas2r136 R	CCTTAATGTGGGTTGAAGCAC
Tas2r143 F	CATTGGCCTCTATGTTGCAG
Tas2r143 R	TGTCCGGTTCCTCATCCA
Gnao1-B F	GCCAAATGCTTGTAGGGGTC
Gnao1-B R	TGAGGCAACCAGACTGGGGT
Gnb1 F	CCTGGACATGGCAAAGAGAATACAG
Gnb1 R	AGGTGAAAAGGGTACAGGGTGCAG
Gnb5 F	CTCGTGTAGATATGACTTCTCCATGAG
Gnb5 R	ACACTGGACGGGGTGAGTGAGTG
Gnb13 F	ATGGAGGAGTGGGATGTGC
Gnb13 R	GCTCGGGGATGGTCTTGGACG
Tas2r114-F	CGGCTGCCACTCACTTATC
Tas2r114-R	CAGCACTTTAATAGTTGCAGTATCATT
GNAT3 -F	GAGAGCAAGGAATCAGCCAG
GNAT3 -R	GTGCTTTTCCCAGATTCACC
GNAI2-F	AGTGCCCTCTGGAGAGACTCG
GNAI2-R	GGCCAATCCGGTCCAAGTTAT
Plcb2-R	TGCTGGATGTCAGATGGTTGCC
Plcb2-F	CGTGCTTTAGGAGGTAGCCACT
